# Effect of intergenerational educational mobility on health of Indian women

**DOI:** 10.1371/journal.pone.0203633

**Published:** 2018-09-07

**Authors:** Akanksha Choudhary, Ashish Singh

**Affiliations:** SJM School of Management, Indian Institute of Technology Bombay, Mumbai, Maharashtra, India; ESIC Medical College & PGIMSR, INDIA

## Abstract

This study aims to analyse the relationship between intergenerational educational mobility and the overall health of the Indian women. It uses a nationally representative survey, India Human Development Survey (IHDS) 2011–12, and logistic regressions to study this relationship. The sample comprises of women aged 45 years and older. We find that the women experiencing upward intergenerational educational mobility (vis-a-vis their mothers) have significantly higher chances of experiencing good overall health compared to the women who are having same or lesser level of education as that of their mothers. Besides, women suffering from short term or major morbidity have remarkably lower chances of having overall good health. Also, women from rural India have significantly lesser chances of having overall good health as compared to that of urban areas. Further, Muslim women have lesser chances of having overall good health as that of women from other religious categories. Moreover, there is a significant variation in the overall health of women as we move from the eastern region to the western region of India.

## Introduction

Education not only plays a vital role in the social and economic development of an economy but also leads to healthier lives for individuals. Health and education have always been among the prime concerns of policymakers as well as researchers for the overall development of a society. It is commonly accepted that better education leads to better paying jobs hence a better lifestyle, which eventually impacts health positively. Therefore, there is a substantial and persistent interdependence between education and health. Extensive researches have been carried out to study the association between the above two [1–10].

The association between education and health of individuals is one aspect of the research on education and health whereas; the education of one generation affecting the education and health of the next generation is another. That said past studies have linked education with reduced risk of illness, increased vitality, longevity, and better school success for future generations [11]. While linking the education of one generation to the education and health related issues of the next generation; the existing studies have impressed upon the fact that it is the mother’s education which plays a more significant role in affecting the educational and health outcomes of children, especially the daughters in both developed as well as developing countries [12–25].

Though the scholarship on relating the educational attainment of one generation to the educational attainment of previous generation (typically called intergenerational educational mobility) is far and wide [26], the relationship between education of one generation and the overall health of the next generation (especially daughters) after completing 14 years of age remains under studied. A few studies and their findings worth noting in this regard are: Park et al. (2013) [12] found that the maternal education in Canada is significantly associated with the mental health of the children in the early adulthood which is independent of paternal education; also offspring of mothers with less than secondary school education had higher odds of major depressive episodes relative to those whose mothers had more education; Marmot et. al. (2010) [27] found that socioeconomic status of individuals measured in terms of parental background greatly impacts the mental health as well as the overall health of the individuals in the United Kingdom; and similarly, Stansfeld et al. (2011) [28] based again on the United Kingdom found that the children who have lower socioeconomic position (measured in terms of parental characteristics) in a society are at a higher risk to experience mid-life depression and other health ailments due to anxiety.

To continue the narrative about the relationship between educational attainment of parents (especially mothers) and the educational achievement as well as overall health status of children (say above 14 years) further; the literature relating how a child has performed vis-a-vis her/his mother in terms of education and her/his overall health is extremely rare. That is, how intergenerational mobility in education (with respect to mother) affects the overall health of a child (once the child has achieved near-adulthood and adulthood) has been extremely under-researched. There are reasons to believe that intergenerational educational mobility might affect the overall/mental health of grownup children. For example, Ward et al. (2016) [29] using data from the Niños Lifestyle and Diabetes Study, assessed the influence of intergenerational educational mobility on depressive symptoms among 603 Mexican-origin individuals and found that sustained stress from downward intergenerational education mobility (individuals having lower educational attainment than parents) leads to depression as well as adversely affects mental condition. Tooth and Mishra (2013) [30] investigated how attaining a level of education that was lower, equivalent to or higher than their parents influenced the mental health of young women in Australia. The study used longitudinal data and three cohorts of women aged 18 years to 75 years in the analysis and found that the downward educational mobility was significantly associated with poorer mental health in the women. Also, Gall et al. (2010) [31] studied the impact of intergenerational educational mobility on the health of Australian adults. The study showed that the children with upward intergenerational educational mobility have a healthier lifestyle and thus better overall health as compared to that of children with downward intergenerational educational mobility. Further, HSBC (2015) [32] covering sixteen countries across the globe reports that parents and people especially in the Asian countries (particularly in India) expect very highly in terms of education from the children up to a point where it begins a burden and leads to severe stress. Also, not attaining a level of education equivalent to that of the parents often leads to social stigma and stress persisting over time which in turn leads to depression.

Having said that, researchers have also manifested that in the coming years depression is going to take the front seat among all health burdens, also the lifetime prevalence of major depressive disorder is estimated to be between 10% and 20% [33, 34]. In 2012, 350 million people were suffering from depression related disorders across the world. Also, by 2030, depression will be the most leading contributor to world disease burden [35]. Further, it has been established that mental health impacts the overall health of the individuals [36]. Moreover, between men and women, it is the women who are at a higher risk than men when it comes to depressive illnesses [37, 38].

Given this context, we in this paper examine the association between intergenerational mobility in education and the overall health status of women in India. We have chosen Indian women for the present study because of multiple reasons: First, India has shown impressive economic growth in the last three decades but the economic growth has not converted into desirable improvements in the economic and social welfare of the masses on one hand and has given rise to widening socioeconomic inequalities on the other [39]; second, India suffers from severe gender based discrimination in different spheres of life as well as Indian women suffer substantially from domestic violence [40–42]; last but not the least, India is among one of the countries where children are expected very highly in terms of education up to a point where it begins a burden and leads to severe stress [32].

We have used logistic regressions and have obtained data from the nationally representative India Human Development Survey 2011–12 for this study. The details of the data and the methods have been described in the next section.

## Data and methods

### Ethics statement

The data were analysed anonymously, using publicly available secondary data; therefore, no ethics review is required for this work.

### Data

The data is taken from the India Human Development Survey (IHDS) 2011–12. As described in detail previously [43], this survey is a nationally representative, multi-topic survey of 42,152 households across India. These households are spread across 33 states and union territories, 384 districts, 1420 villages and 1042 urban blocks located in 276 towns and cities. The survey covers all states and union territories of India with the exception of the islands of Andaman/Nicobar and Lakshadweep. Two one-hour interviews in each household covered health, education, employment, economic status, marriage, fertility, gender relations, and social capital. The IHDS was jointly conducted by the University of Maryland and the National Council of Applied Economic Research (NCAER), New Delhi [44]. In addition to the household and individual questionnaires, IHDS 2011–12 includes village, school, and medical facility surveys. Extensive Census data were also merged for contextual analyses at the village, district, and state levels. Within the household survey, several sections also focused on the household’s connections to the wider community [43, 44].

As mentioned previously [43], the survey was carried out in face-to-face interviews in seven modules covering (1) socio-economic condition of the household including income, employment, educational status, consumption expenditure, and social capital; (2) interview with one ever-married woman aged 15–49 years per household was conducted regarding the health, education, fertility, family planning, marriage, and gender relations; (3)interview with youths in the households aged 15–18 years was also conducted regarding education, employment, marriage, life skills, future planning, friendship and risky confidential behaviours; (4) further, short reading, writing, and arithmetic knowledge tests for children aged 8–11 and youths aged 15–18 years in the household were administered; (5) moreover, height and weight measurement of children under age 5, aged 8–11, their mothers, and other available household members were conducted; (6) in addition, facilities assessment of one government and one private primary school as well as a primary health care facility in the community was conducted; (7) furthermore, village questionnaire assessing employment opportunities and infrastructure facilities in the village was administered [44].

The survey instruments were translated into thirteen Indian languages and were administered by local interviewers. The fieldwork was carried out by twenty collaborating institutions under the supervision of the National Council of Applied Economic Research (NCAER), New Delhi. Fieldwork began in November 2011 and was almost completed by October 2012 [43, 44]. The 15–49 years aged ever married women (one from each household) from the 42, 152 households form the sample for analysis in this paper. Information regarding their schooling and schooling of their mothers has been used for generating the estimates of intergenerational educational mobility presented in this paper. The total sample size comprises of 39,395 women from every nook and corner of India. The actual sample is smaller than 42,152 because some households did not have any ever-married woman in the age group 15–49 years, in addition, those ever-married women who were studying at the time of the survey are not included in the analysis sample. Appropriate sampling weights have been used to derive the estimates at national and regional levels. The details of the sampling weights are available in the survey report [43, 44].

### Methods

#### Variables

Outcome Variable: The outcome variable for the current study is the self-reported overall health of the women. Often researchers have a view that self-reported health is a very subjective measure [45] and may lead to various response biases, such as, self-reporting is likely to get affected by individual’s perception and interpretation of symptoms and the readiness to report illness in interview [46]; and cultural differences in readiness to report illness [47]. However, another school of thought believes the self-reported health as the right way to measure the health of a person as it not only captures biological or physical health but also mental or psychological health of the person [48, 49]. Of late, the above mentioned debate is shifting towards self-reported health being a better indicator of overall health status, for example, see Schnittker and Bacak [50] (and the references therein) which argues that self-reported overall health has better predictive validity.

In IHDS, an eligible woman (an ever married woman in the age group of 15–49 years) from every household was asked to rate her overall health status, as she perceives it to be. The response was captured as one of the following: ‘very good’, ‘good’, ‘fair’, ‘bad’ and ‘very bad’. For the purpose of analysis and ease of interpretation, we have regrouped the above five categories into two. That is, the responses of the women who answered ‘very good’ and ‘good’ were clubbed under ‘good’ and the rest were regrouped under ‘not good’.

Main Independent Variable: The intergenerational educational mobility of the Indian women is the main independent variable of interest in the current study. There are three different categories for this variable. These are (1) ‘No mobility’ (when daughter and mother have exactly same years of education); (2) ‘Downward mobility’ (when daughter’s years of schooling are less than that of her mother); (3) ‘Upward mobility’ (when daughter’s years of schooling are more than that of her mother).

Control Variables: Several control variables have also been used in the analyses. These control variables have been arrived at by their relevance to the women’s overall health and their use in the earlier studies [1–3, 11, 30, 51–53] on the subject. The control variables used in the present study have been segregated into three levels, i.e., individual level, household level and community level.

*Individual level variables*:(1) Short term morbidity (categorical; yes/no): This variable captures whether the woman has suffered from fever, cough or diarrhoea during the last 30 days prior to the survey; (2) Major morbidity (categorical; yes/no): This variable captures whether the woman has suffered from any of the following long term illnesses–cataract, tuberculosis, high-BP, heart diseases, diabetes, leprosy, cancer, asthma, polio, paralysis, epilepsy, mental illness, STD/AIDS or any other long term illness during the last 12 months prior to the survey; (3) Self-employment status (categorical; unemployed/employed); (4) Age and Square of age (continuous): the square of age takes care of the non-linearity between the health outcome and age of the women.

*Household level variables*:(1) Employment status of spouse (categorical; unemployed/employed); (2) Children below six years of age (categorical; yes/no): Whether there are children below six years in the household; (3) Household per capita income (continuous): In Indian Rupees per month; (4) Caste (categorical–Scheduled Castes (SC), Scheduled Tribes (ST), Other Backward Castes (OBC) and Other Castes (OC)): The above categorization is a meaningful representation of the Indian social fabric along caste lines [25, 54]; and (5) Religion (categorical): Religion has been categorized into five categories, namely Hindu (the majority religious group), Muslim (the largest group among religious minorities), Christians, Sikhs and Others.

*Community level variables*: (1) Regions (categorical): We have grouped the different states and Union Territories (UTs) of India into five regions, namely: (1) North, (2) Central, (3) East, (4) West and (5) South; (2) Location (categorical; Urban and Rural).

#### Regression strategy

We have used logistic regression to model the relationship between the self-reported overall health status of the Indian women and intergenerational educational mobility. The overall health of the Indian women is the outcome of interest along with intergenerational educational mobility as the main independent variable of interest and some relevant control variables as mentioned in the earlier section. There is always a possibility of a high degree of correlation between several control variables as well as the main independent variable. Hence, we have checked for correlations among all the variables. We also performed tests for multicollinearity (Variance Inflation Factor (VIF) test as well as Condition Index test proposed by [55], but found no evidence of any substantial or significant multicollinearity among the independent variables included in the models.

## Results

### Descriptive statistics

#### Distribution of females for all-India, urban and rural areas

Table 1 presents the distribution of the sample by various individual, household and community level variables. The table presents the above for the all-India level as well as for the urban and rural areas.

**Table 1:** Distribution of Women by all India, urban and rural.

	All India	Urban	Rural
**Health (%)**			
Good health	77.80	82.04	75.82
Not Good health	22.20	17.96	24.18
**Mobility (%)**			
No Mobility	41.38	26.13	48.48
Downward mobility	2.61	3.05	2.41
Upward Mobility	56.01	70.82	49.11
**Short term morbidity (%)**			
No	80.44	83.89	78.82
Yes	19.56	16.11	21.18
**Major morbidity (%)**			
No	91.68	92.16	91.46
Yes	8.32	7.84	8.54
**Employment (Self) (%)**			
Unemployed	59.34	77.19	51.02
Employed	40.66	22.81	48.98
**Age (Mean)**	36.00	37.03	35.59
Up to 20 years	5.02	3.06	5.94
21–30 years	33.60	30.57	35.01
31–40 years	34.59	36.65	33.62
Above 40 years	26.79	29.71	25.43
**Employment (Spouse) (%)**			
Unemployed	7.64	8.72	7.14
Employed	92.36	91.28	92.86
**Kid/s below 6 years (%)**	41.31	36.77	43.43
**Per Capita Income (Annual, Mean)**	28096.32	39371.75	22220.43
**Caste (%)**			
Other Castes (OC, General)	27.36	35.13	23.73
OBCs	42.60	43.47	42.19
SCs	22.14	18.68	23.76
STs	7.90	2.72	10.32
**Religion (%)**			
Hindu	82.70	78.18	84.81
Muslims	12.48	16.50	10.61
Christians	1.89	2.68	1.52
Sikhs	1.17	1.03	1.24
Others	1.76	1.62	1.83
**Region (%)**			
Northern region	12.91	14.64	12.10
Central region	23.82	17.54	26.75
Eastern region	25.76	16.23	30.20
Western region	14.90	21.44	11.85
Southern region	22.61	30.14	19.09

*Sample sizes*—(All India-38,238; Urban-13,100; Rural-25,138)

*Source*: Authors’ computations based on the data IHDS 2011–2012.

Some crucial findings from Table 1 are as follows: about 77 percent of females for all India have good health however; this proportion is much higher in urban areas which is 82 percent and marginally lower in rural areas which is about 75 percent. Also, this might be a case of presence of better health facilities in urban areas for women as compared to that of rural areas [56]. About 56 percent of daughters have experienced upward intergenerational education mobility for all India; this proportion is as high as 70 percent for urban areas and about 49 percent for rural areas. Also, this disparity in intergenerational educational mobility between urban and rural areas is in line with the results of Choudhary and Singh (2017) [26].

Nearly 19 percent of the surveyed women had fever, cough or diarrhoea (short term morbidity) during the last 30 days prior to the survey for all India, where as this proportion is slightly higher in rural areas which is about 21 percent. One of the important reasons for this higher proportion can be lack of proper sanitation in rural India [57]. Further, about 8 percent of women at the all India level had suffered from cataract, tuberculosis, high-BP, heart diseases, diabetes, leprosy, cancer, asthma, polio, paralysis, epilepsy, mental illness, STD/AIDS or any other long term illness during the last 12 months prior to the survey (major morbidity); there are not any notable variations in this proportion as we move from rural to urban areas.

As we move to the employment status of the women at the all India level, about 40 percent of women are employed but there is a vast difference in this proportion when we compare rural and urban areas. In rural areas about 49 percent of women are employed while for urban areas this proportion is as less as 23 percent. This humongous difference can be a result of a large proportion of women being working as farmers and agricultural labourers in rural India, whereas in urban areas there has been a significant decline in labour force participation among women because of rising educational enrolment of young women and lack of appropriate employment opportunities [58–60]. Also, the average age of the women in the present study is 36 years with about 33 and 34 percent of women being in the age group of 21–30 years and 31–40 years, respectively. Moreover, about 92 percent of women have their husbands employed and it doesn’t vary much between rural and urban areas. Further, about 41 percent of women have at least one child below the age of six years at the all-India level. Furthermore, the average per capita income is Rs. 28096 for all India and is significantly higher in the urban areas (Rs. 39371) compared to the rural areas (Rs 22220).

While looking at the caste distribution of Indian women, an important point to observe here is that ST women contribute only 2.7 percent to urban women population whereas this proportion is 10 percent in rural areas. This vast difference has also been explained in Hall and Patrinos (2012) [61]. Coming to the regional distribution of women, it can be seen that only 16 percent is contributed by women from the eastern region to the urban population of women whereas the contribution of the women from the same region to the rural population of women is about 30 percent. On the other hand, about 12 percent of rural women are from the western region whereas about 21 percent of urban women are from the western region.

#### Distribution of Females Having Good Health by All India, Urban and Rural Areas

Table 2 presents the distribution of the women with overall good health by the various individual, household and community level variables. The table presents the above for the all-India level as well as for the urban and rural areas, separately.

**Table 2:** Prevalence of women (percentage) with good overall health: By socioeconomic characteristics.

	All India	Urban	Rural
**Mobility**			
No Mobility	73.24	76.22	72.54
Downward mobility	74.62	78.34	72.00
Upward Mobility	81.30	84.34	79.24
**Short term morbidity**			
No	80.16	84.16	78.15
Yes	68.04	70.72	67.08
**Major morbidity**			
No	80.32	84.14	78.5
Yes	49.94	56.84	46.95
**Employment (Self)**			
Unemployed	77.98	82.19	74.99
Employed	77.51	81.52	76.66
**Age**			
Up to 20 years	82.69	84.66	82.26
21–30 years	82.24	85.62	80.81
31–40 years	77.68	82.71	74.99
Above 40 years	71.44	77.24	68.46
**Employment (Spouse)**			
Unemployed	77.64	83.02	75.26
Employed	77.81	81.94	75.85
Kid/s below 6 years	80.44	84.79	78.69
No Kid below 6 years	75.93	80.43	73.59
**Caste**			
Others (General)	78.39	82.88	75.28
OBCs	78.18	82.07	76.21
SCs	75.34	80.67	73.48
STs	80.53	80.04	80.79
**Religion**			
Hindu	78.00	82.04	76.30
Muslims	74.08	80.00	69.37
Christians	81.54	87.42	76.75
Sikhs	87.03	86.56	87.28
Others	84.15	90.74	81.42
**Region**			
Northern region	74.86	79.12	72.47
Central region	76.20	73.75	76.80
Eastern region	69.61	77.69	67.68
Western region	89.63	91.42	88.35
Southern region	82.66	91.42	81.89

*Sample sizes*—Women with good health-29,343.

*Source*: Authors’ computations based on the data IHDS 2011–2012.

Some stylized findings from Table 2 are as follows: women with upward intergenerational educational mobility contribute the highest proportion to the total women with overall good health; the trend is similar for rural as well as urban areas. Similarly, women who didn’t suffer from any minor (short term morbidity) or major illnesses (major morbidity) have higher proportion of women with overall good health at the all-India level and for both urban and rural areas. About 78 percent of the unemployed women have reported of having overall good health whereas 77 percent of working women feel that they have overall good health at the all India level; the trend is similar in urban areas with slightly higher proportion of women having overall good health for both the cases of employed and unemployed, whereas in rural areas proportion of women having overall good health is slightly lower than that of urban areas for both the cases. Also, a higher proportion of employed women compared to the unemployed ones have reported overall good health in rural areas.

Further, it can be observed from the Table 2 that as the age increases the proportion of women reporting overall good health goes down gradually. Moreover, employment status of spouse doesn’t make much of a difference in proportion of women reporting overall good health at the all-India level as well as for rural areas, however in urban areas when the spouse is not working a higher proportion of women have reported of having overall good health. The reason for this can be–when husbands aren’t working either they are financially well to do or they help their wives in managing the household chores and eventually reducing the burden on the women [62].

In addition, about 80 percent of women having children below six years of age report of having overall good health as compared to about 76 percent of women reporting of overall good health with no child below six years of age. This could be possible because of the observation that early motherhood brings with it subjective wellbeing for the mother and also when the children grow up mothers’ age also increases thus reducing the proportion of women with overall good health [63].

While analysing the distribution of women having overall good health by their castes; an interesting thing to note is that the proportion of Scheduled Tribe women reporting overall good health is highest among all the caste groups and it is about 80 percent in urban as well rural areas. Also, among the religions, the proportion of women reporting overall good health remains lowest for the Muslim women. Besides the proportion of women reporting overall good health remains lowest for the eastern region (which comprises of the economically and demographically poorer states of India, such as Bihar, Jharkhand, Orissa, West Bengal etc.) and highest for western region which comprises of the economically and demographically advanced states of India, such as, Gujarat and Maharashtra.

### Multivariate results

The main results from the multivariate analysis presented in Table 3 are as follows: Firstly, it can be seen from Table 3 that at the all-India level, the odds of having overall good health for women increases significantly (1.11 times) when they have better education as that of their mothers as compared to the women who have same level of education as that of their mothers. In urban areas, in case of upward intergenerational educational mobility women have even higher (significant) chances of having overall good health (1.22 times) as compared to the women who have same level of education as that of their mothers. In rural areas also, the odds of having overall good health for a woman increases (1.07 times) when she has better education as compared to that of her mother.

**Table 3:** Odds of having good overall health by Indian women by their socioeconomic characteristics.

	All India	Urban	Rural
**Intergenerational educational mobility; Reference: No mobility**			
Downward Mobility	0.9229	1.0067	0.8942
	(0.0754)	(0.1352)	(0.0925)
Upward Mobility	1.1146***	1.2204***	1.0748**
	(0.0315)	(0.0627)	(0.0365)
Short term morbidity	0.6360***	0.6432***	0.6316***
	(0.0192)	(0.0355)	(0.0229)
Major morbidity	0.3110***	0.3056***	0.3131***
	(0.0119)	(0.0199)	(0.0148)
**Self-employment; Reference: Unemployed**			
Employed	0.9240***	1.0333	0.8651***
	(0.0260)	(0.0560)	(0.0290)
**Spouse's employment; Reference: Unemployed**			
Employed	1.0015	0.9724	1.0575
	(0.0473)	(0.0735)	(0.0644)
Per capita income	1.0000***	1.0000***	1.0000
	(0.0000)	(0.0000)	(0.0000)
Age	0.9944	0.9899	0.9980
	(0.0103)	(0.0197)	(0.0122)
Square of age	0.9998	0.9998	0.9997
	(0.0001)	(0.0003)	(0.0002)
**kid below age of 6 years; Reference: No kid below 6 years**			
At least one kid below age of six years	1.0025	1.0246	0.9932
	(0.0338)	(0.0639)	(0.0400)
**Castes; Reference: Others (general)**			
OBCs	1.0084	0.9650	1.0533
	(0.0325)	(0.0527)	(0.0425)
SCs	0.9696	0.9365	1.0087
	(0.0367)	(0.0624)	(0.0469)
STs	1.1889***	0.8210	1.3112***
	(0.0652)	(0.1049)	(0.0823)
**Religion; Reference: Hindu**			
Muslims	0.8487***	0.9139	0.8564***
	(0.0335)	(0.0573)	(0.0443)
Christians	1.0607	1.0803	1.1030
	(0.0946)	(0.1553)	(0.1260)
Sikhs	2.4771***	1.8508***	2.7052***
	(0.2497)	(0.3936)	(0.3106)
Others	1.0539	1.2931	0.9660
	(0.1272)	(0.2774)	(0.1422)
**Region; Reference: Northern Region**			
Central region	1.2106***	0.9342	1.3085***
	(0.0465)	(0.0655)	(0.0602)
Eastern region	0.8454***	0.9797	0.7719***
	(0.0313)	(0.0640)	(0.0352)
Western region	3.0866***	2.8896***	3.2043***
	(0.1671)	(0.2738)	(0.2118)
Southern region	1.5815***	1.4273***	1.6714***
	(0.0632)	(0.0973)	(0.0830)
**Area; Reference: Rural area**			
Urban	1.2137***		
	(0.0359)		
Constant	4.9375***	6.3356***	4.5327***
	(0.9832)	(2.4507)	(1.0656)
Observations	38,238	13,100	25,138

Notes

*** p<0.01

** p<0.05

* p<0.1

*Sample size*: All India-38,238; Urban-13,100; Rural-25,138

*Source*: Authors’ computations based on the data IHDS 2011–2012.

Secondly, short term morbidity significantly reduces the odds (about 0.63 times) of having overall good health for women at the all-India level, urban areas as well as rural areas. Even in the case of major morbidity the odds (about 0.30 times) of having overall good health for women are drastically low for all India, urban area as well as rural areas. The above findings are in line with our expectation.

Thirdly, employment status of a woman does make a difference in her chances of experiencing overall good health. It can be seen from the Table 3 that the odds (0.92 times) of having overall good health reduces significantly when women are employed as compared to the unemployed women. This is the case for women from rural areas because most of the women in rural areas are involved in occupational activities which are physically tiring and then they also have to also do the household chores which eventually lead to a poorer reported health of those women.

Fourthly, other control variables such as age, square of age, employment status of spouse, children below six years of age do not affect the overall health of women significantly. However, as it can be seen from the Table that the ST women, especially in rural areas, have significantly higher odds (1.31) of having overall good health as compared to the women belonging to the OC category. This may be the case because of the fact that the health captured here is self-reported health and mostly ST community in the context of rural areas in India are physically and socially isolated and typically stay away from the villages (have their own physically and socially isolated hamlets) and hence may not have the awareness about good health. Also, STs have a history of staying in forests and away from civilizations and don’t judge their health in a way similar to a person with access to modern medicine [64].

Another thing to note here is that the Muslim women have significantly lower chances of having overall good health in rural as well as urban areas as compared to that of Hindu women or the women belonging to the other religions. The Sikh women have the highest odds (2.47) of having overall good health as compared to that of women of any other religion. This might be because Sikhism as a religion has an element of healthier and happier lives [65]; but this result needs further investigation which can be taken as future research.

In addition, there is a massive disparity as we move from the eastern region to the western region when it comes to odds of having overall good health among women. Women from the western region (which comprises of the economically and demographically the most advanced states, such as Maharashtra and Gujarat) have highest odds (3.08 times when northern region is taken as reference) of having overall good health. On the other hand, women from eastern region (which comprises of the economically and demographically poorer states of India, such as Bihar, Jharkhand, Orissa, West Bengal etc.) have the lowest odds (0.84) for having overall good health.

Finally, women from urban areas have higher odds of having overall good health as compared to that of women from rural areas because of better sanitation, education and healthcare facilities [57, 66]. Also, in rural areas the prevalence of gender based inequality in various social and demographic indicators as well as gender based domestic violence are relatively higher compared to urban areas [67, 68]; further, incidence of poor nutritional status is also higher in rural areas. These all could be other important factors for lower odds of having overall good health in rural areas. In addition, urban women have more autonomy and decision making power in early seeking treatment for their health problems which could also be a possible reason for higher odds of having overall good health in urban areas compared to rural areas.

We now present our main conclusions along with some discussion on the main findings, thus concluding the paper.

## Conclusions and discussion

Using a nationally representative survey, our paper analyses how attaining a particular level of education in the Indian context by a woman which is equal, less or more than her mother impacts the overall health of that woman. The paramount finding from the study is that women who have better education than that of their mothers in terms of years of education have significantly higher chances of having overall good health. As the health measured in this study is self-reported overall health, it captures the psychological component of health as well. As expected, women having short term morbidity or major morbidity have lesser chances of having overall good health. Besides, women who have been employed especially in rural areas have lesser odds of experiencing overall good health. Surprisingly it has been observed that women from scheduled tribe have higher odds of having overall good health. Furthermore, Muslim women have significantly lower chances of experiencing overall good health. There has been a considerable increase in odds of having overall good health for women as we move from the east to the west with the eastern region having lowest odds and the western region having the highest odds for having overall good health. Also, women from urban areas have higher odds of experiencing overall good health compared to the rural women.

The results from the current study support the positive association between upwards intergenerational educational mobility and overall health in Indian women. This finding is in line with those found in the case of mostly developed countries; for example, Tooth and Mishra (2013) [30] found that downward educational mobility was significantly associated with poorer mental health in the women in Australia. Also, Gall et al. (2010) [31] showed that the Australian children with upward intergenerational educational mobility have healthier lifestyle and thus better overall health as compared to that of children with downward intergenerational educational mobility. Further, Ward et al. (2016) [29] in the North American context found that sustained stress from downward intergenerational education mobility (individuals having lower educational attainment than parents) leads to depression as well as adversely affects mental condition. Our study is perhaps the first study to examine the link between intergenerational educational mobility and the health status of women in the developing countries in general and India in particular.

One finding of our study which is rather surprising is that the women from the Scheduled Tribes (STs) have higher odds of having overall good health compared to the women belonging to the Other Caste (OC) groups. This is surprising, because STs have historically suffered from severe discrimination and social as well as physical isolation in India and significantly lag behind the OCs (the ‘General’ or ‘Upper’ Castes) in different indicators of economic, social and demographic welfare including public health [54, 39, 69–71]. As mentioned earlier, the women from the Scheduled Tribes (STs) having higher odds of having overall good health compared to the women belonging to the Other Caste (OC) groups may be because of the fact that the health captured in the present paper is self-reported health and the ST community mostly stay away from the villages (have their own physically and socially isolated hamlets) and hence may not have the awareness about good health. Also, STs have a history of staying in forests and away from civilizations and don’t judge their health in a way similar to a person with access to modern medicine [64]. Moreover, the deplorable condition of Muslim women as far as health is concerned is in line with the findings of earlier studies, the most recent one being [72]. In addition, the great regional divide as far as the overall health of the Indian women is concerned is also not surprising and adds to the existing narrative of socioeconomic disparities between the demographically, economically and socially advanced regions of west and south and the demographically, economically and socially disadvantaged regions of central and east [73–75].

As the key finding from the current study indicates that women who have better education as compared to that of their mothers have better overall health suggests that the policymakers need to plan and take actions to improve the educational status of women in India. It also focuses on the importance of special efforts, which needs to be put in, to educate the girls whose mothers have lower levels of education else they can be trapped in low educational endowment trap over generations and won’t be able to break this trap [26]. The governmental programs for female children, such as, ‘Kanya Saaksharta Protsahan Yojna’**, ‘**Kasturba Gandhi Balika Vidyalaya Yojna’ and ‘Ballika Samridhi Yojana’ are good initiatives in this regard. Another important issue which needs to be addressed is the drastic difference in the overall health of women from rural and urban areas which indicates the need for better sanitation and healthcare facilities in rural areas. Once again the Government of India’s health care programs for rural women, such as, ‘Janani Suraksha Yojana’ and ‘Adolescent Reproductive Sexual Health’ under the Rural Health Mission are good schemes in this context.

As the measure of health used in the present study is self-reported health, our study suffers from a limitation that along with the legitimate differences in health of the women there might be a few differences associated to the knowledge of existing conditions or awareness about own health among the women. However, most of the studies investigating the relationship between health and education have used self-reported health as the self-reported health is generally considered one of the best ways to measure health because it also takes into account the mental health. Moreover, it is also argued that it is the individual who is in a much better position (than anybody else) to judge her/his overall health [49]. Besides, the existing scholarship also shows the prevalence of using a multi-point scale extensively to measure self-reported health as used in the current study [76–78]. Finally, the recent studies have found that the predictive validity of self-rated health is increasing over time ([50] and the references therein).

Last but not the least; one important way to carry this research agenda forward will be to look into how intergenerational educational mobility affects mental health in the Indian context.
